# Supplementary material for: Benefits of Collisional Cross Section Assisted Precursor Selection (caps-PASEF) for Cross-linking Mass Spectrometry
Source: Mol Cell Proteomics. 2020 Nov 25;19(10):1677–87. doi: 10.1074/mcp.RA120.002094 (PMC8015012; doi:10.1074/mcp.RA120.002094)
Supplement: Supplementary file 1 [file mmc1.docx]

**Benefits of Collisional Cross Section Assisted Precursor Selection (caps-PASEF) for Cross-linking Mass Spectrometry**

Barbara Steigenberger^1,2,4^, Henk W.P. van den Toorn^1,2^, Emiel Bijl^1,2^, Jean-François Greisch^1,2^, Oliver Räther^3^, Markus Lubeck ^3^, Roland J. Pieters^4^, Albert J.R. Heck^1,2^, Richard A. Scheltema^1,2$^

1. Biomolecular Mass Spectrometry and Proteomics, Bijvoet Center for Biomolecular Research and Utrecht Institute for Pharmaceutical Sciences, Utrecht University, Padualaan 8, 3584 CH Utrecht, The Netherlands.
2. Netherlands Proteomics Centre, Padualaan 8, 3584 CH Utrecht, The Netherlands.
3. Bruker Daltonik GmbH, Fahrenheitstrasse 4, 28359 Bremen, Germany
4. Department of Chemical Biology & Drug Discovery, Utrecht Institute for Pharmaceutical Sciences, Utrecht University, 3508 TB Utrecht, The Netherlands

$ Address reprint requests to R.A. Scheltema, Padualaan 8, 3584 CH Utrecht, The Netherlands, [r.a.scheltema@uu.nl](mailto:r.a.scheltema@uu.nl)

**Running title:** The timsTOF Pro applied to XL-MS

**Supplementary Table S1 – RAW files used in this study.** File-type ‘mgfmeta’ contains CCS, mobility and intensity. This is linked to file-type ‘mgf’ on scannumber/first scan. Files ending with _PSMS.txt contain the Mascot results and files ending with _CSMS.txt contain the XlinkX results.

|  | **Associated files** | **Type** | **Panels** | **Comments** |
| --- | --- | --- | --- | --- |
| **Fig. 1,2,S3, S4** | BSAPhoX60minlike60_Slot1-1_01_3657.d  BSAPhoX60minlike60_Slot1-1_01_3657.mgf  BSAPhoX60minlike60_Slot1-1_01_3657.mgfmeta  BSAPhoX60minlike60_Slot1-1_01_3657_PSMs.txt  BSAPhoX60minlike60_Slot1-1_01_3657_CSMs.txt  BSAPhoX60minlike60_polygon_Slot1-1_01_3669.d  BSAPhoX60minlike60_polygon_Slot1-1_01_3669.mgfmeta  BSAPhoX60minlike60_polygon_Slot1-1_01_3669_PSMS.txt  BSAPhoX60minlike60_polygon_Slot1-1_01_3669_CSMS.txt | PASEF  caps-PASEF | **1B-E,**  **S3A-C**  **S4A,B**  **1B-E, 2A-E,**  **S4A,B** | This set of files contains acquisition on BSA from which the polygon for the further runs is based. |
| **Fig. 3** | ProteinMixPhoX70minlike60_Slot1-3_01_3671.d  ProteinMixPhoX70minlike60_Slot1-3_01_3671.mgf  ProteinMixPhoX70minlike60_Slot1-3_01_3671.mgfmeta  ProteinMixPhoX70minlike60_Slot1-3_01_3671_CSMs.txt  ProteinMixPhoX70minlike60_Slot1-3_01_3671_PSMs.txt  ProteinMixPhoX70minlike60_polygon_Slot1-3_01_3672.d  ProteinMixPhoX70minlike60_polygon_Slot1-3_01_3672.mgf  ProteinMixPhoX70minlike60_polygon_Slot1-3_01_3672.mgfmeta  ProteinMixPhoX70minlike60_polygon_Slot1-3_01_3672_CSMs.txt  ProteinMixPhoX70minlike60_polygon_Slot1-3_01_3672_PSMs.txt | PASEF  caps-PASEF | **3A-D** | This set of files contains the acquisition on our protein mix. |
| **Fig. 4** | HeLaPhoX150minlike60_16MSMS_Slot1-4_01_3676.d  HeLaPhoX150minlike60_16MSMS_Slot1-4_01_3676.mgf  HeLaPhoX150minlike60_16MSMS_Slot1-4_01_3676.mgfmeta  HeLaPhoX150minlike60_16MSMS_Slot1-4_01_3676_PSMs.txt  HeLaPhoX150minlike60_16MSMS_Slot1-4_01_3676_CSMs.txt  HeLaPhoX150minlike60_polygon16MSMS0_75_Slot1-4_01_3677.d  HeLaPhoX150minlike60_polygon16MSMS0_75_Slot1-4_01_3677.mgf  HeLaPhoX150minlike60_polygon16MSMS0_75_Slot1-4_01_3677.mgfmeta  HeLaPhoX150minlike60_polygon16MSMS0_75_Slot1-4_01_3677_PSMs.txt  HeLaPhoX150minlike60_polygon16MSMS0_75_Slot1-4_01_3677_CSMs.txt | PASEF  caps-PASEF | **4A-D** | This set of files contains the acquisition on HeLa. |
| **Fig. S1** | 20190520_AAAAKAAAAAR_726_10-90eV_d3-110_PASEF.d  20190520_AAAAKAAAAAR_726_10-90eV_d3-110_PASEF_CSMs.txt  20190520_AAAAKAAAAAR_1089_10-90eV_d3-110_PASEF.d  20190520_AAAAKAAAAAR_1089_10-90eV_d3-110_PASEF_CSMs.txt | PASEF direct infusion | **S1A-C** | Initial definition of the CE calibration curves |
| **Fig. S2** | BSAPhoX60min20_Slot1-2_01_3659.d  BSAPhoX60min20_Slot1-2_01_3659.mgf  BSAPhoX60min30_Slot1-2_01_3661.d  BSAPhoX60min30_Slot1-2_01_3661.mgf  BSAPhoX60min40_Slot1-2_01_3663.d  BSAPhoX60min40_Slot1-2_01_3663.mgf  BSAPhoX60min50_Slot1-2_01_3665.d  BSAPhoX60min50_Slot1-2_01_3665.mgf  BSAPhoX60min60_Slot1-2_01_3667.d  BSAPhoX60min60_Slot1-2_01_3667.mgf  BSAPhoX60min70_Slot1-2_01_3668.d  BSAPhoX60min70_Slot1-2_01_3668.mgf  BSAPhoX60min80_Slot1-2_01_3666.d  BSAPhoX60min80_Slot1-2_01_3666.mgf  BSAPhoX60min90_Slot1-2_01_3664.d  BSAPhoX60min90_Slot1-2_01_3664.mgf  BSAPhoX60min100_Slot1-2_01_3662.d  BSAPhoX60min100_Slot1-2_01_3662.mgf  BSAPhoX60min110_Slot1-2_01_3660.d  BSAPhoX60min110_Slot1-2_01_3660.mgf  BSAPhoX60min120_Slot1-2_01_3658.d  BSAPhoX60min120_Slot1-2_01_3658.mgf  csmtable.txt  csmtable.mgf | PASEF fixed collision energies ranging from 20 to 120 in steps of 10 | **S2** | This set of files contains the acquisitions on BSA with fixed collision energies acquired in scrambled order.  The CCS values were read directly from the MGF file (in the header: TITLE). The file csmtable.txt contains the identifications linked to the scannumbers and csmtable.mgf all spectra extracted from all mgf file. |


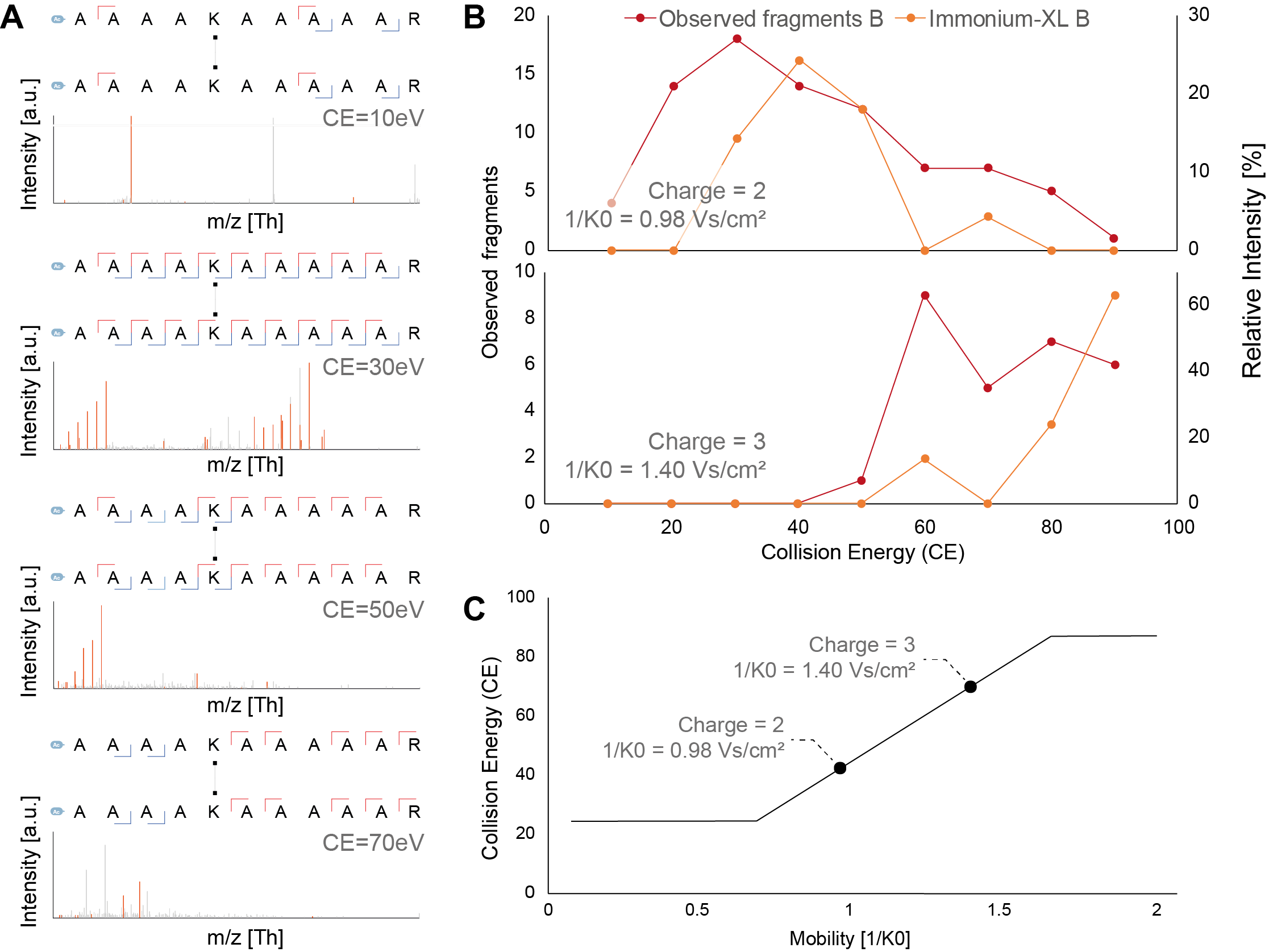


**Supplementary Figure S1 – Collision energy optimization on the cross-linked peptide dimer Ac-AAAAKAAAAAR. (A)** Fragmentation spectra for the cross-linked peptide dimer at charge state 2 at different collision energies. At low collision energies, no fragments are produced, which only start to appear at energies that are more elevated. At energy levels higher than the optimal, over-fragmentation starts to occur, visible here in the disappearance of fragments linking an intact peptide to the other, fragmented, peptide. **(B)** Expressing the quality of the fragmentation spectra at various collision energies in observed fragments (equates directly to sequence coverage) and relative intensity of cross-linking reagent specific immonium ion (see Steigenberger *et al*(1)) the optimal points for charge state 2 is found at ~ 40 eV and for charge state 3 at ~70 eV. **(C)** Final calibration curve linearly extrapolating based on the two observations and limiting the energy at 25 eV, below which typically no fragmentation is observed, and at 85 eV, above which typically only over fragmentation is observed.


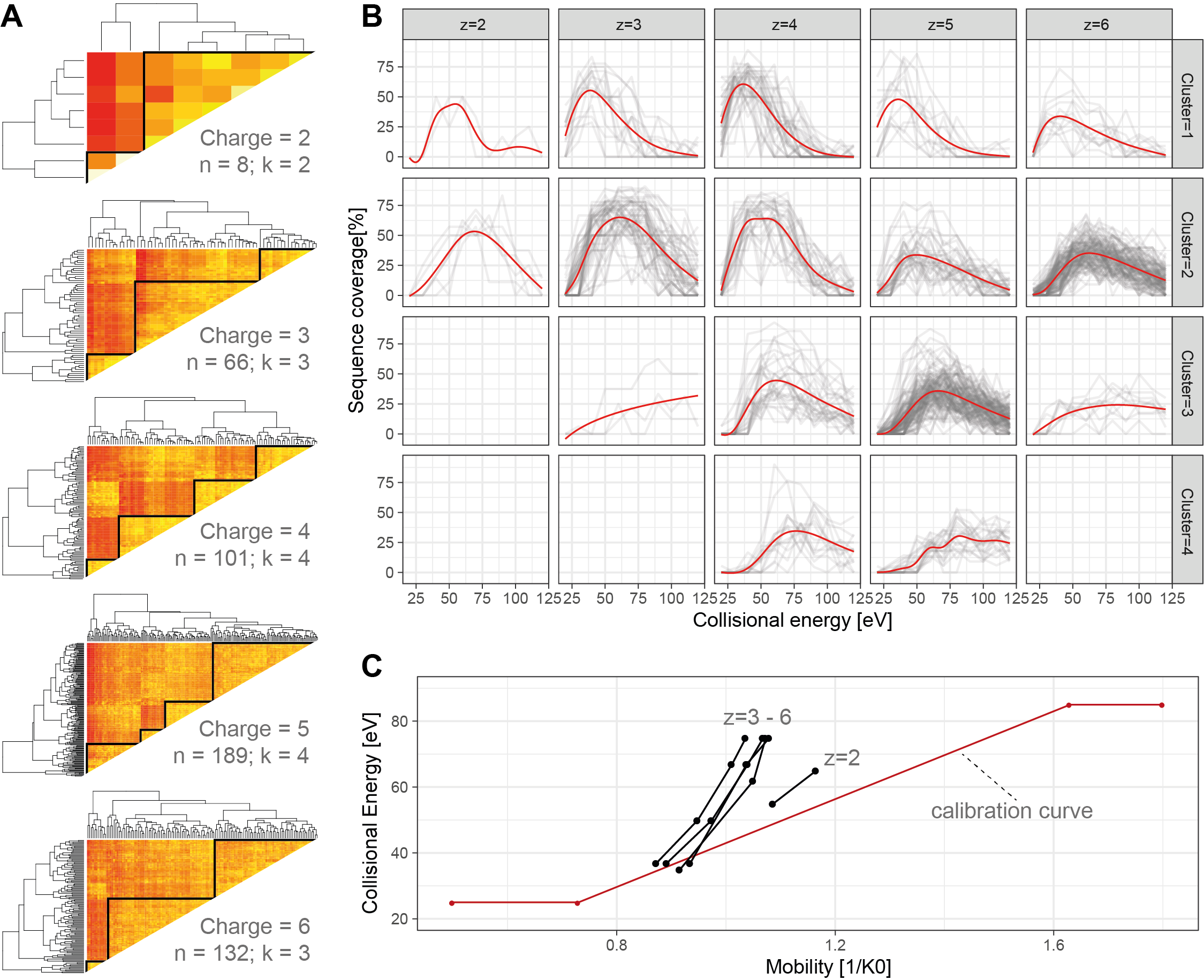


**Supplementary Figure S2 – Collision energy optimization on BSA with fixed collision energies. (A)** After extraction of identifiable spectra (no FDR control was applied, spectra for collisional energies where the identification could not be made were located by correlating the spectra with precursor mass within 20 ppm and retention time within 2 min), for all spectra the sequence coverage was calculated – resulting in a sequence coverage trace over all the collisional energies. By correlating each trace against all other traces for each charge state independently, we were able to construct dendrograms from which matching traces could be selected in an unbiased fashion. **(B)** Extraction of the clusters shows that the unsupervised clustering method extracts sequence coverage traces with similar properties and that the apex of the average trace (indicated by the red line) slowly shifts to higher collisional energies for each cluster. **(C)** Plotting the apex collisional energy for the median mobility of each cluster shows that the initially determined calibration curve for lower mobilities fits well for charge state 3 - 6, although for higher mobilities starts to diverge from the calibration. For charge 2 we observer divergent behaviour from the rest of the charge states, suggesting that altering the calibration curves in the instrument control software could potentially have a beneficial effect on the identification performance. As the control software does not at the point of writing not support the complex behaviour as extracted by this analysis, we opted to keep the initially extracted calibration curve as a conservative estimate that fits well with the lowest charge state.


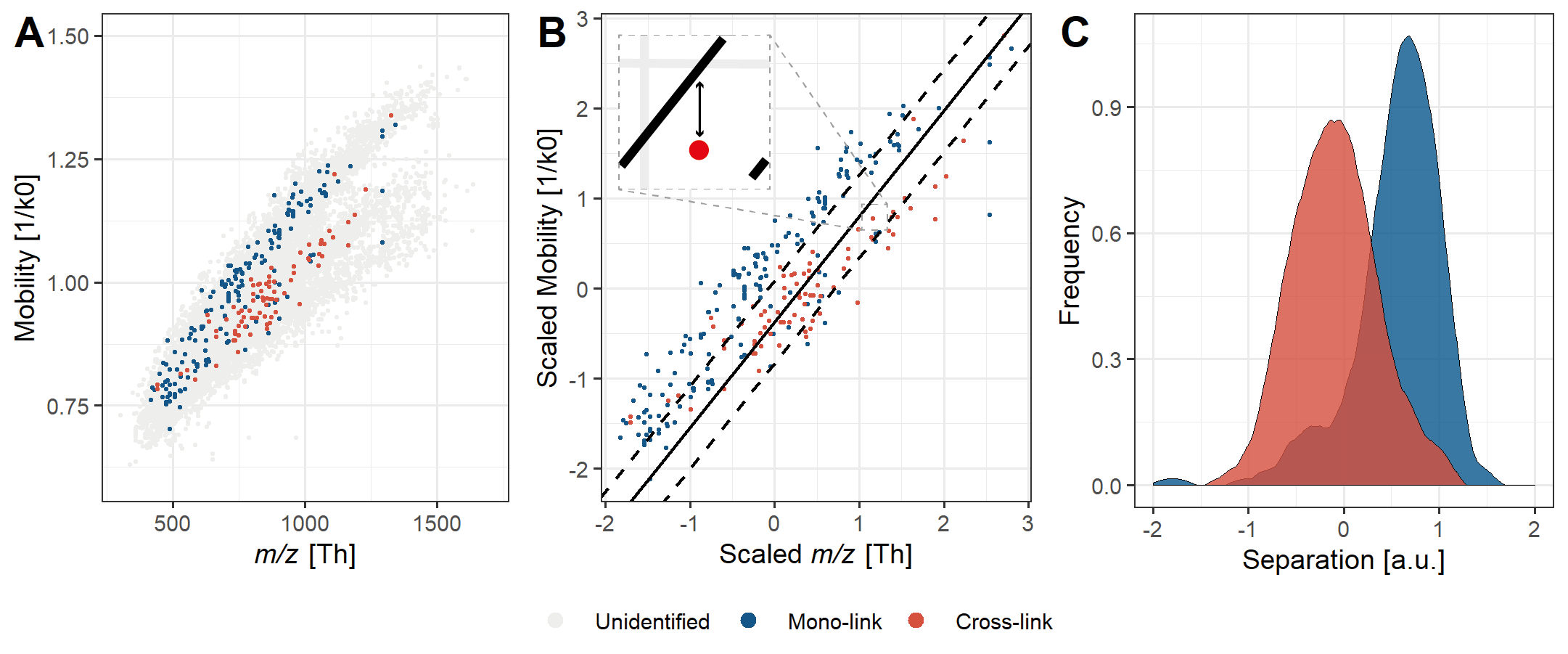


**Supplementary Figure S3 – Estimation of physical separation in mobility space for the BSA run in PASEF mode. (A)** The m/z (Th) versus Mobility (1/k0) for all classes of molecules. **(B)** After removal of the unidentified molecules and z-score scaling of the values a linear support vector (SVM) was fit, optimized on separating between the peptides/mono-links and the cross-links, in R with the package ‘e1071’. The solid line denotes the separating plane fit by the SVM and the dotted lines the confidence interval. **(C)** The mobility distance (*i.e.* on the y-axis alone) of each identification to the solid line is calculated and expressed in the density plot for both peptide/mono-link and cross-link identifications.

**Supplementary Table S2 – The polygon definition for our caps-PASEF experiments.**

| **Mono-isotopic mass** | **CCS** |
| --- | --- |
| 2000 | 500 |
| 2000 | 750 |
| 2500 | 1000 |
| 5000 | 1200 |
| 6000 | 1100 |
| 4400 | 750 |
| 3000 | 500 |


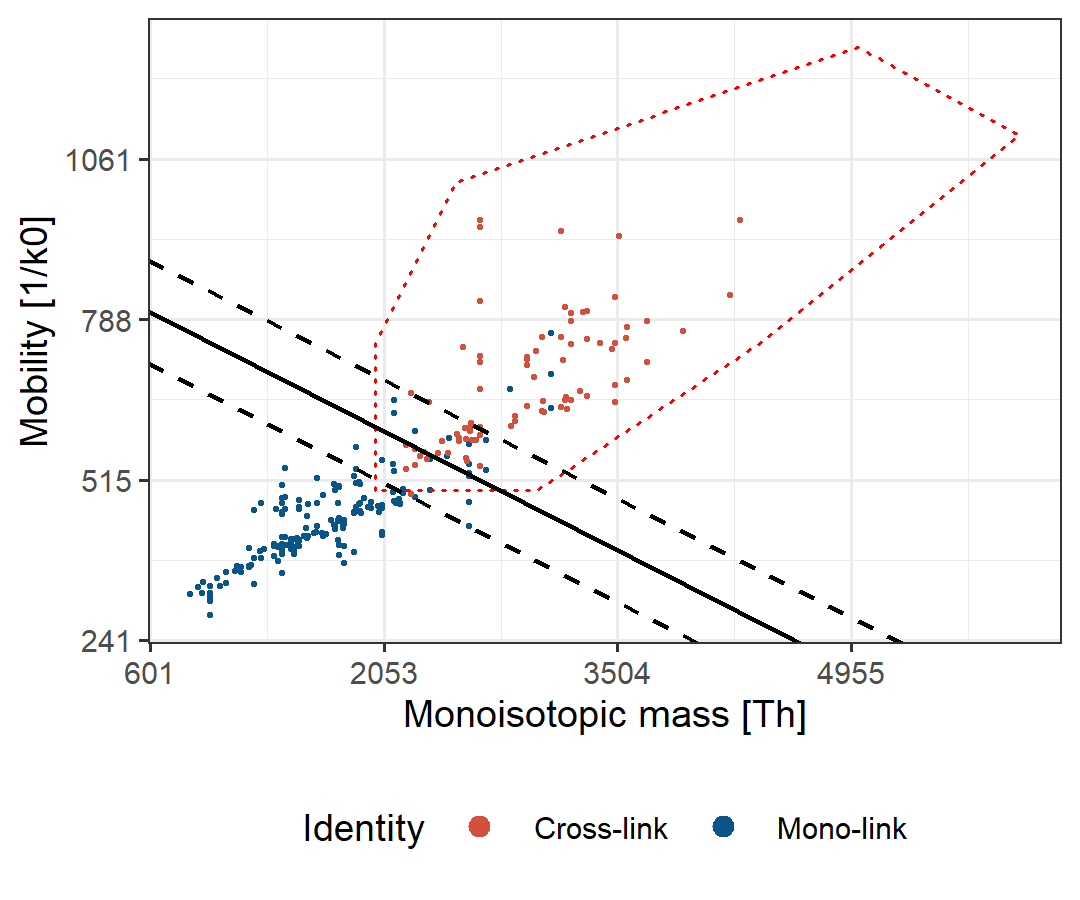


**Supplementary Figure S4 – Estimating the boundary between cross-links and mono-links for the polygon definition.** A linear SVM model was fit to the BSA data recorded in PASEF mode to estimate the edge between the two classes of molecules. To be conservative, the bottom-left edge of the polygon was selected on top of the left decision boundary of the SVM model, which indicates the area below which the SVM classification is certain the identification is a mono-link.


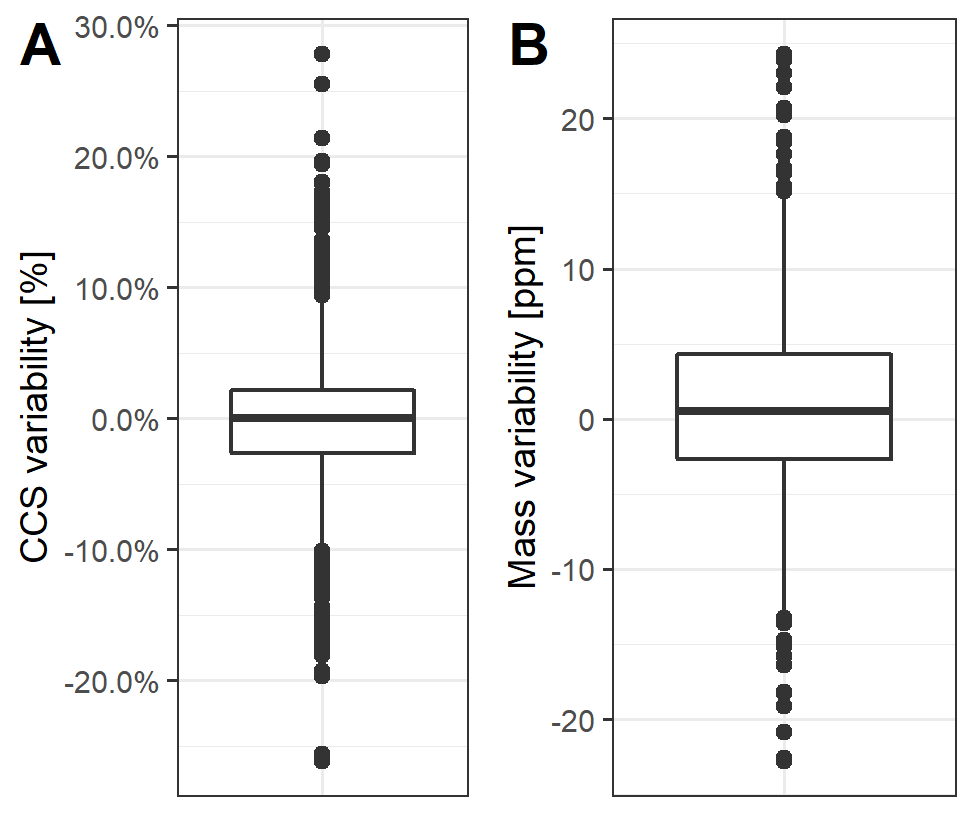


**Supplementary Figure S5 – Inter-measurement variability for the protein-mix. (A)** Detection of CCS values for identified cross-linked peptides shows the TIMS device has a precision of +/- 10% over two measurements for the vast majority of the cases, with 50% of the detections within +/- 2.5%. **(B)** Deviations between the detected mono-isotopic masses of the two measurements confirms that the instrument measures with an accuracy of +/- 20 PPM.


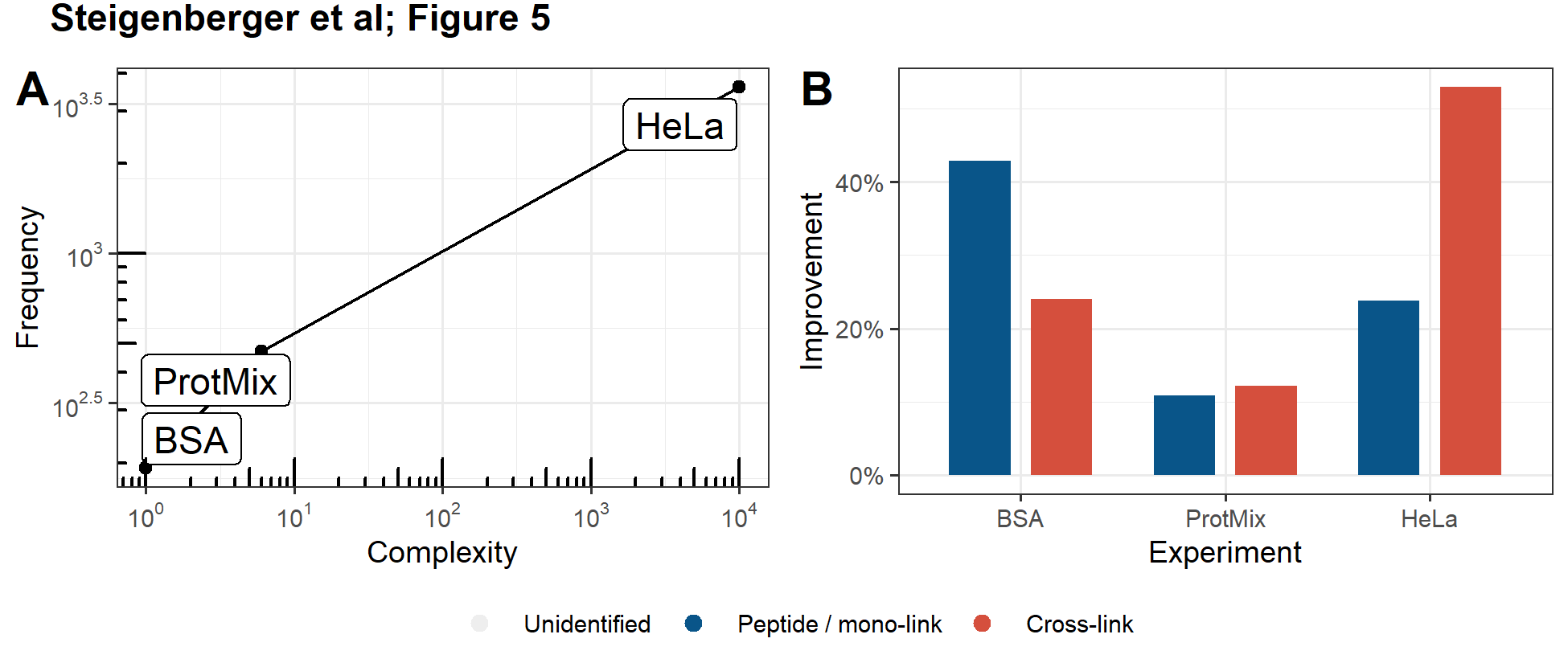


**Supplementary Figure S6 – Performance over increasing complexity. (A)** Number of identified peptides / mono-links per sample. To obtain a better overview of the amount of peptide/mono-links for the different sample complexities we plotted the detected amounts versus the expected complexity with an estimated protein count of 10,000 for the HeLa sample. From this visualization it is clear that with the rising complexity the possibilities for creating mono-links increase logarithmically. As such, it is not surprising that the benefit capsPASEF is not greater than the 10% found for this dataset as the cross-linked peptides are obfuscated by the peptides/mono-links. **(B)** Identification improvements inside the polygon region. When considering solely the identifications inside the polygon however shows that the benefit of capsPASEF is substantial for both peptide/mono-link as well as cross-link identifications. Surprisingly, for the BSA dataset a large benefit is accomplished inside the polygon with ~40% for the mono-link/peptide identifications and ~20% for the cross-linked peptides. This is likely a result of more acquired ions due to focusing the mass spectrometry leading to higher quality fragmentation spectra. For the higher complexity samples, the benefit for the cross-link identifications becomes more pronounced than for the peptide/mono-link identifications with the biggest benefit for the high complexity sample. This opens the possibility of true gas-phase fractionation, whereby the same sample is reanalyzed with focusing the mass spectrometer on different sections of the mobility.

**References**

1. Steigenberger, B. A., Schiller, H. B., Pieters, R. J., and Scheltema, R. A. (2019) Finding and using diagnostic ions in collision induced crosslinked peptide fragmentation spectra. *Int. J. Mass Spectrom.* 444, 116184
